# Population Statistics for 1895

**Published:** 1897-05-01

**Authors:** 


### The Hospital

A Journal of

The Medical Sciences and Hospital Administration.

Vol. XXII.—No. 553.] [May 1, 1897.

### POPULATION STATISTICS FOR 1895.

Somewhat tardily in relation to the period of occurrence of the events recorded, and even some months later than was formerly customary, the Registrar-General has just issued his report on the births, deaths, and marriages of 1895. We accept the official order in which these several incidents of human life are arranged; although it is not at first sight clear why marriages should have what may be called a "post-mortem" position. Whatever may be the reason for this, there can be no doubt as to the propriety of giving the first place to births; and, in view of the colonising propensities of English people, and of the new spheres which are opening for their activity in various parts of Africa and elsewhere, it is highly satisfactory to record that nearly a million of new lives (922,291, composed of 468,886 males and 453,405 females) were added to the population in the course of the year. This represents 30·4 births to every thousand persons living, and is 0·8 per thousand below the average of the preceding ten years, a diminution which will perhaps not be wholly objectionable in the eyes of domestic economists, while at the same time it is not sufficiently considerable to occasion any alarm lest we should be beginning to tread in the footsteps of France, and to become dependent upon immigrants for the supply of workers in important industries. As is only too certain to be the case, the total number of births is increased by no less than 38,836 that were illegitimate; and, as is also usual, some counties continue to occupy so bad a pre-eminence, in relation to this question, that the social conditions conducive to illegitimacy seem to demand the earnest attention of their leading inhabitants. Taking England and Wales, the illegitimate births were in the proportion of 42 per thousand of the total; but they were as low as 29 per thousand in Middlesex and Essex, 31 in Monmouthshire, 33 in South Wales, and 35 in Somerset and Warwick; while in Hunts they were 63, in Norfolk 64, in North Wales 65, in Cumberland 70, in Hereford 72, and in Shropshire 74. We chance to have before us some corresponding figures for the year 1886; and, although there is a slight general improvement, it is remarkable how little the proportions have changed. In 1886, the number of illegitimate births per thousand was 32 in the extra-metropolitan portion of Middlesex, 34 in Essex, and 38 in London, against 72 in Norfolk, 75 in North Wales, 79 in Cumberland, 80 in Hereford, and 82 in Shropshire. It will be observed that the rates in Norfolk, North Wales, Hereford, Cumberland, and Shropshire were then and are now nearly double the average of the kingdom, and more than double the averages of some other counties; so that the facts call eloquently for investigation by local social reformers, and for endeavours to bring about a better state of things.

The marriages, as might be expected from the prosperity of the country, show a tendency to increase above the low average of the decennium 1885-94; but the increase is at present only small in amount, and does not quite keep up with that of 1894; while the percentages of widows and widowers desirous of resuming the chains of matrimony have slightly declined.

It could hardly be expected that the death-rate of 1895 could be maintained at the low point which it touched in 1894, but the increase which has occurred, and which brings it up to 18·7 per thousand persons living, is still 0·2 per thousand below the average rate of the last decennium. The lowest total death-rate was furnished by the counties of Rutland and Westmorland, where the figures were 14·2; and the highest was by Lancashire, where they were 22·3. Among the causes there was no remarkable variation from recent proportions, except in the circumstance that the certified cause in twenty instances was hydrophobia, a larger number than has been recorded in any year since 1889. The victims in 1895 were 17 males and three females.

A new table is given with the report, which brings out in a very curious way the undeserved stigma which may have been attached to certain metropolitan districts, with reference to particular causes of death, on account of charitable institutions which those districts contain. Thus, the effect of the Hospital for Consumption, aided to some extent by that of the extra-parochial Marylebone Infirmary, is to raise the mortality from phthisis in Kensington to 2·35 per thousand; while, if the deaths occurring in these institutions are referred to the districts which furnished the patients, the proper mortality of Kensington falls to 1·42, or scarcely more than half what it would at first sight appear. The phthisis mortality of Guy's Hospital acts in a similar way, and raises that of St. Olave's parish to a fraction more than three per thousand above its proper rate. It is clear that persons in search of salubrious London localities must not omit this element in the case from their calculations, and that before they condemn any district on the score of a high mortality from this or the other cause, they must satisfy themselves whether its reputation may not be suffering wrongfully by reason of its hospitals.

